# Suturing fragmented landscapes: Mosaic hybrid zones in plants may facilitate ecosystem resiliency

**DOI:** 10.1073/pnas.2410941122

**Published:** 2025-07-28

**Authors:** Rob Massatti, Trevor M. Faske, Ivana M. Barnes, Elizabeth A. Leger, Thomas L. Parchman, Bryce A. Richardson, L. Lacey Knowles

**Affiliations:** ^a^United States Geological Survey, Southwest Biological Science Center, Flagstaff, AZ 86001; ^b^Department of Ecology and Evolutionary Biology, Museum of Zoology, University of Michigan, Ann Arbor, MI 48104; ^c^Department of Biology, University of Nevada, Reno, NV 89557; ^d^United States Department of Agriculture Forest Service, Rocky Mountain Research Station, Moscow, ID 83843

**Keywords:** mosaic hybrid zone, seed-based restoration, climate change, genotype-environment association, range dynamics

## Abstract

Many widespread plant taxa of western North America have diversified into phenotypically and genetically divergent lineages due to complex biogeographic histories across heterogeneous landscapes. Mosaic hybrid zones can form when geographically co-occurring, yet environmentally distinct, lineages cross-pollinate and form hybrids that occupy unique environmental niches absent of a geographic cline. This expands the total environmental space across which parental and hybrid individuals grow, resulting in larger, less fragmented geographic distributions. Here, we highlight hybridization mosaics across three study systems containing taxa critical to widespread plant communities in western North America: *Ericameria nauseosa*, *Artemisia tridentata*, and *Sphaeralcea fendleri*. The systems contain diverged taxa that co-occur across the landscape and hybridize readily. Hybridization among taxa has facilitated niche expansion into intermediate environments consistent with unique combinations of adaptive genetic variation, creating more continuity within each study system—study systems occupy ~820 to 270,000 km^2^ more geographic area by virtue of hybridization. Furthermore, hybrids are predicted to play important roles in future climates, as they may occupy 8 to 475% larger distributions compared to present. Convergent patterns signal mosaic hybridization as an underappreciated mechanism with broad ecological and evolutionary ramifications. Leveraging mosaic hybridization may assist the creation of restoration management plans that aim to mitigate the deleterious effects of habitat fragmentation on ecosystems in the context of climate change.

## Significance

Mosaic hybrid zones offer opportunities for closely related taxa to occupy a larger environmental niche, which in topographically and environmentally heterogeneous regions may mean a larger, less fragmented geographic distribution. We quantify the ecological impact of mosaicism in three study systems important to restoration and landscape management across western North America by predicting the distributions of parents and hybrids across present and future environments. Land disturbance and climate change reinforce the necessity of developing strategies to create resilient plant communities, and hybrids that suture together parental distributions by occupying intermediate environmental space may present novel management opportunities by integrating speciation science with conservation practice.

Plant populations and species that diversify during their biogeographic histories often hybridize in areas of secondary contact (1). Mosaic hybrid zones form when hybridization between divergent evolutionary lineages results in hybrid genotypes that maximize fitness in intermediate or novel environments absent of a geographic cline (2, 3). Parental lineages and hybrids together may be more continuously distributed due to the occupation of a wider environmental niche compared to what would be realized by the parental taxa alone (4–6). In turn, less fragmented distributions, especially for foundational taxa, have the propensity to facilitate community resilience and stability (7, 8). Hybrids may also act as conduits of adaptive genetic variation between parental lineages (9, 10) or as reservoirs of novel genetic variation that aid population persistence (11). In the context of rapid climate change and broadscale habitat disturbance (12), the ecological and evolutionary ramifications of mosaic hybrid zones may be critical to understand for maintaining diverse ecosystems and their services (13).

With declining biodiversity (14, 15) and increasingly fragmented ecosystems (16), attention to restoring degraded habitat has gained global attention (17, 18). However, management strategies are challenged by climate change, which causes restoration failures (19) and confounds evidence-based guidance (20, 21). To buffer against uncertainty and bolster positive outcomes, there is active development of novel methods of restoration (22, 23), tools to assist seed transfer (24–26), and research to ascertain local adaptation and the outcomes of assisted migration (27–29). For taxa that form mosaic hybrid zones (30, 31), unique combinations of adaptive variation may make hybrids well suited to navigate the complexities of large-scale and rapidly changing environmental conditions (11), in addition to facilitating expanded distributions across environmental gradients. Opportunities to leverage the adaptive flexibility of these systems (i.e., parental taxa plus hybrids) in management efforts necessitate a deeper understanding of the extent and consequences of mosaic hybridization.

Foundational species or clades with large and environmentally complex distributions often have complicated biogeographic histories and phenotypic diversity recognized as taxonomic variation. We leverage three suites of such taxa known to hybridize and that are critical components of plant communities in western North America: *Ericameria nauseosa* (Pall. ex Pursh) G.L. Nesom & G.I. Baird (rubber rabbitbrush; Asteraceae), *Artemisia tridentata* Nutt. (big sagebrush; Asteraceae), and *Sphaeralcea fendleri* A. Gray (Fendler’s globemallow; Malvaceae). *E. nauseosa* and *A. tridentata* are foundational shrub species spanning the entirety of western North America that exhibit intraspecific phenotypic and taxonomic variation associated with environmental variation and hybridization (32, 33). Specifically, we focus on *E. nauseosa* subsp. *nauseosa* (hereafter *E. n. nauseosa*) and *E. nauseosa* subsp. *consimilis* (Greene) G.L. Nesom & G.I. Baird (hereafter *E. n. consimilis*) following (34) and diploid *A. tridentata* subspecies including *A. tridentata* subsp. *tridentata* (hereafter *A. t. tridentata*) and *A. tridentata* subsp. *vaseyana* (Rydb.) B. Boivin (hereafter *A. t. vaseyana*). *S. fendleri* is an ecologically important forb occupying disturbed soils across the western drylands. Because *Sphaeralcea* species readily hybridize and can be difficult to identify due to intermediate morphologies (35), we included species in close geographic proximity to *S. fendleri* that occasionally overlap morphologically, including *S. angustifolia* (Cav.) G. Don, *S. hastulata* A. Gray, and *S. incana* Torr. ex A. Gray. All the above taxa are critical to restoration efforts across dryland habitats in western North America. For more information, see *SI Appendix*, Supplemental Text**.

To investigate the ecological and evolutionary ramifications of mosaic hybrid zones, we first use genomic data to identify divergent evolutionary lineages (hereafter parental taxa) and hybrids among them. Next, we assess correlations between allele frequencies and environmental gradients that may be indicative of adaptation and use machine learning algorithms to predict ancestry based on the environment for each lineage (i.e., parental taxa and hybrids). We leverage relationships between environmental characteristics and ancestry to project parental and hybrid climate envelopes across geographic space and quantify the ecological ramification of hybridization for each study system in both present and future climates (i.e., the change in area occupied by the study system resulting from hybridization). Our research leverages data and methods described in ref. 32, which we expand upon to highlight the convergence of mosaicism across study systems. Finally, we synthesize the evolutionary and ecological consequences of hybridization in the context of habitat fragmentation and landscape management efforts.

## Results

### Divergent Evolutionary Lineages and Readily Distinguished Hybrids.

**Fig. 1.**
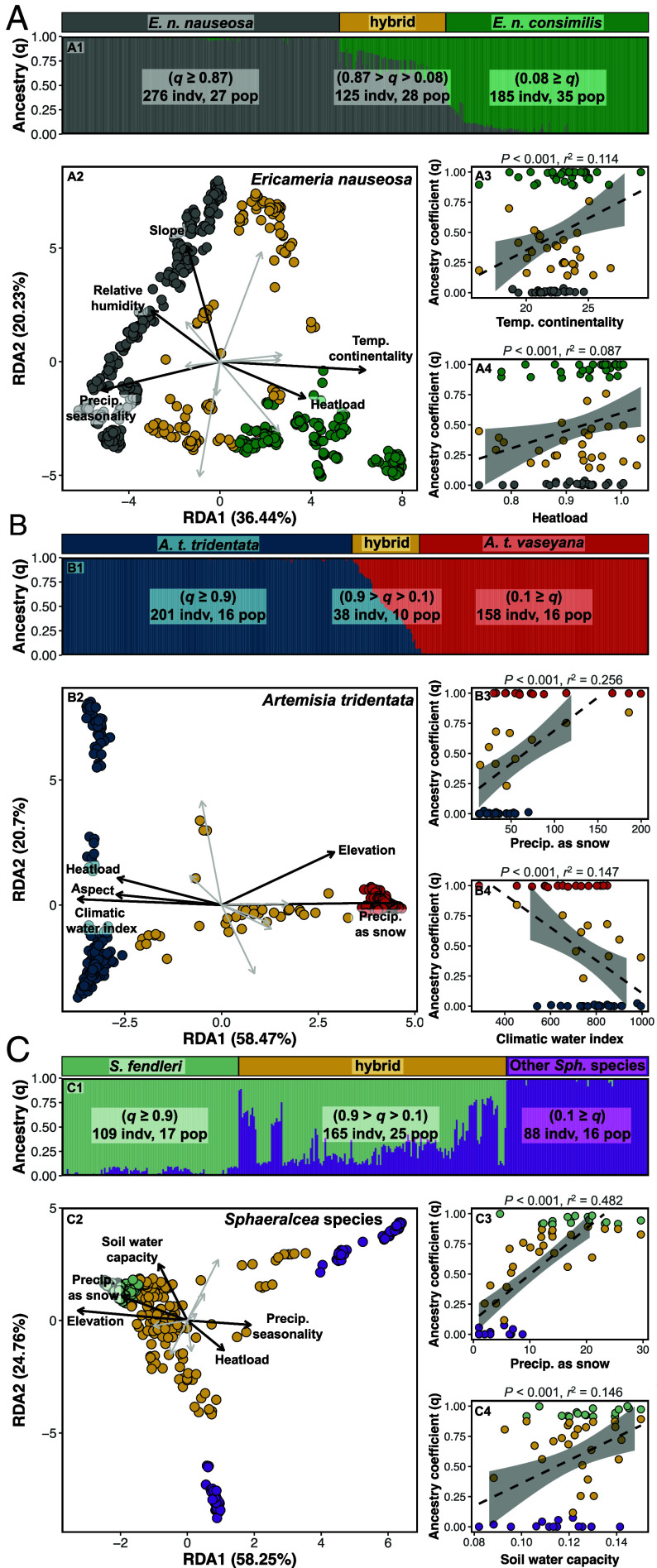
Each study system consists of genetically distinct lineages (i.e., parental taxa) that co-occur geographically. Hybrids form throughout the ranges of parental taxa and vary considerably in their degree of hybridization. Ancestry class is predicted by environmental conditions, rather than geography (i.e., mosaic hybrid zones). Plots depict genetic variation in (*A*) *E. nauseosa,* with hybrids occurring between the well-supported *E. n. nauseosa* and *E. n. consimilis* subspecies, (*B*) *A. tridentata*, with hybrids occurring between the diploid *A. t. tridentata* and *A. t. vaseyana* subspecies, and (*C*) multiple *Sphaeralcea* species. For each species, numbered subpanels represent the following: 1) Individual ancestry coefficients (*q*) from the hierarchical Bayesian model of entropy for *k* = 2, ordered by the first PCA axis (*SI Appendix*, Fig. S1). Color proportion of each bar depicts individual ancestry proportion. 2) Redundancy analysis (RDA), which illustrates how environmental variation predicts genetic variation between the lineages and hybrids. Black lines and labels denote the top five environmental variables predicting ancestry in a multivariate framework. 3 and 4) The top two environmental predictors of ancestry coefficients (*q*) assessed through univariate beta regressions, with *P*-values and *r*^2^ values reported above each panel.

Based on reduced representation genomic sequencing data, estimated ancestry of individual plants provided unambiguous support for the existence of divergent evolutionary lineages (i.e., the parental taxa) with geographically dispersed sites of hybridization in each of our study systems (Fig. 1 and *SI Appendix*, Figs. S1 and S2). *F*_ST_ and Nei’s *D* are highest in pairwise comparisons between parental taxa and intermediate between each parental taxon and hybrids when assigning individuals as taxon/hybrid (i.e., based on ancestry coefficients rather than collection locality; *SI Appendix*, Fig. S3). *F*_ST_ values remain high when assessing pairwise comparisons between populations of parental taxa, even when they are in close geographic proximity (Dataset S1). Variance partitioning illustrates that environmental variation, conditioned upon geography, is strongly associated with genomic variation, explaining 10.5%, 56.2%, and 30.4% of variation within *E. nauseosa, A. tridentata,* and *Sphaeralcea* respectively (*SI Appendix,* Table S1). An additional 24.5% and 47.9% of genomic variation is confounded between environment and geography for *E. nauseosa* and *Sphaeralcea* such that the relative influence of these factors cannot be disentangled. This demonstrates both the variability in how species are influenced by extrinsic factors and the difficulty implicit in investigating biological processes given complex abiotic covariation, as caused by the topographic and environmental heterogeneity of western North America. Much less genomic variation is associated with geography (i.e., latitude and longitude) after accounting for environmental variation (13.6%, 4.6%, and 0%) for *E. nauseosa, A. tridentata, and Sphaeralcea*, respectively (*SI Appendix,* Table S1).

### Hybrids Occupy Unique Environmental Space and Relationships between Genotypic and Environmental Variation Support Adaptation.

**Fig. 2.**
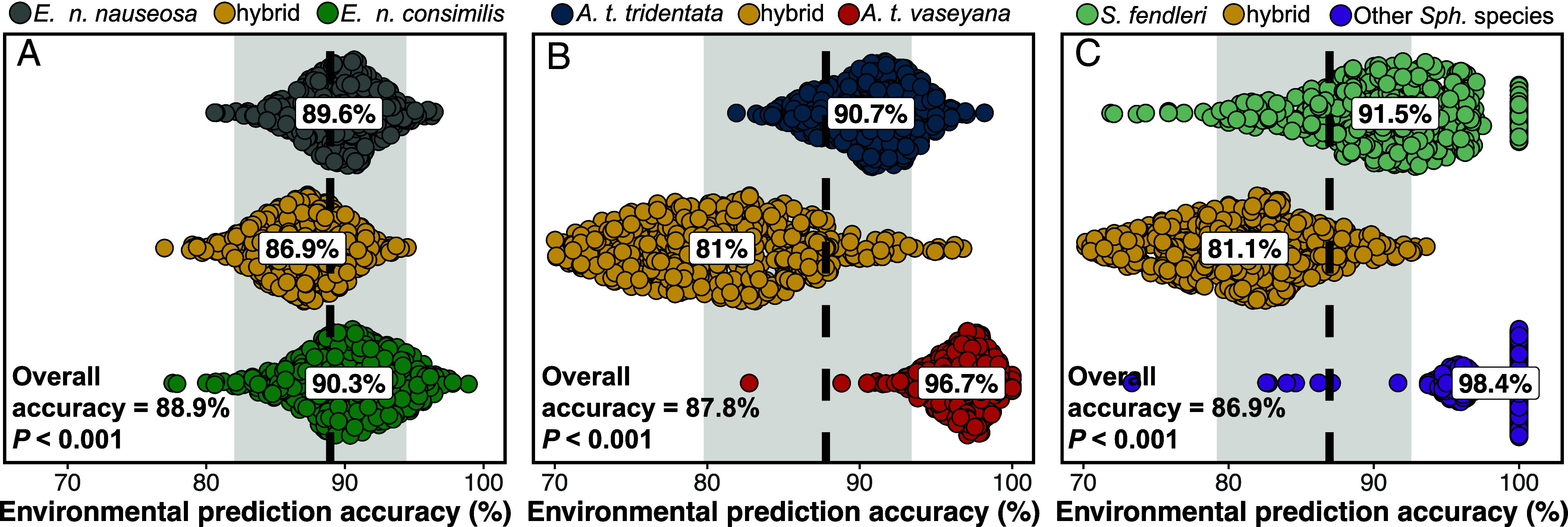
Environmental predictions of ancestry class (i.e., parental taxa or hybrid) for each study system using Random Forest for (*A*) *E. nauseosa,* (*B*) *A. tridentata*, and (*C*) *Sphaeralcea*. Points represent permuted accuracy within each lineage, vertical spread represents point density, and text boxes show mean accuracy percentages. Dashed lines, overall accuracy percentages, and shaded bars represent the overall mean predictive accuracy and the 95% bootstrapped CI. *P*-values were assessed through a randomized permutation test (*SI Appendix,* Fig. S7).

Hybridization in our study systems was expected given previous reports and intermediate morphologies (33–35). Capitalizing on approaches used in ref. 32, we quantify the fidelity of hybrids to intermediate environments using multivariate (redundancy analysis), univariate, and machine learning (Random Forest) models (Fig. 1, Table 1, and *SI Appendix,* Figs. S4–S6 and Table S2). Our results indicate that environmental variation drives the distribution of allele frequencies across the landscape, coinciding with parental and hybrid ancestry. Overlap in environmental predictor variables across the systems, irrespective of the method used, is indicative of shared drivers of environmental niche divergence. For example, heat load was highly ranked across all systems, and precipitation as snow is important for both *A. tridentata* and *Sphaeralcea* (Fig. 1 and *SI Appendix,* Table S2). However, we also identify unique environmental influences for taxa, including temperature continentality for subspecies in *E. nauseosa*, climatic water index for subspecies in *A. tridentata*, and precipitation seasonality for *Sphaeralcea* species. When using only knowledge of environmental variation at the sampling locations, Random Forest models assigned individuals to their correct ancestry class (i.e., parental or hybrid) with high degrees of accuracy (88.9%, 87.8%, and 86.8% for *E. nauseosa*, *A. tridentata*, and *Sphaeralcea*, respectively; Fig. 2 and *SI Appendix,* Fig. S7). Each of these systems represents a mosaic hybrid zone; hybrids occupy unique habitat bridging environmental space between parental taxa, occur at multiple geographically dispersed locations, and environmental variation is a strong predictor of both parental and hybrid ancestries.

**Table 1.** Predicted geographic area across which parental taxa and hybrids are distributed based on Random Forest machine learning models

	Present area (km^2^) (%)	SSP126 area (km^2^) (%)	SSP585 area (km^2^) (%)	SSP126 change from present (km^2^) (%)	SSP585 change from present (km^2^) (%)
*E. nauseosa*					
*E. n. consimilis*	553,229 (49.09)	596,504 (53.85)	590,568 (54.58)	43,275 (7.82)	37,339 (6.75)
*E. n. nauseosa*	95,602 (8.48)	53,527 (4.83)	19,458 (1.80)	−42,075 (−44.01)	−76,144 (−79.65)
hybrid	269,591 (23.92)	263,396 (23.78)	290,099 (26.81)	−6,195 (−2.30)	20,508 (7.61)
≥2 taxa	208,577 (18.51)	194,237 (17.54)	181,862 (16.81)	−14,340 (−6.88)	−26,715 (−12.81)
total	1,126,999 (100)	1,107,664 (100)	1,081,987 (100)	−19,335 (−1.72)	−45,012 (−3.99)
*A. tridentata*					
*A. t. tridentata*	164,771 (43.24)	225,346 (60.84)	287,543 (81.08)	60,575 (36.76)	122,772 (74.51)
*A. t. vaseyana*	212,769 (55.84)	141,421 (38.18)	60,897 (17.17)	−71,348 (−33.53)	−151,872 (−71.38)
hybrid	822 (0.22)	1,357 (0.37)	4,729 (1.33)	535 (65.09)	3,907 (475.30)
≥2 taxa	2,688 (0.71)	2,280 (0.62)	1,472 (0.42)	−408 (−1518)	−1,216 (−45.24)
total	381,050 (100)	370,404 (100)	354,641 (100)	−10,646 (−2.79)	−26,409 (−6.93)
*Sphaeralcea* sp.					
*S. fendleri*	54,962 (25.76)	27,812 (12.94)	14,105 (6.69)	−27,150 (−49.40)	−40,857 (−74.34)
hybrid	54,384 (25.49)	64,266 (29.89)	54,320 (25.76)	9,882 (18.17)	−64 (−0.12)
Other *Sph.* sp.	87,515 (41.02)	98,527 (45.83)	116,737 (55.36)	11,012 (12.58)	29,222 (33.39)
≥2 taxa	16,479 (7.72)	24,399 (11.35)	25,716 (12.19)	7,920 (48.06)	9,237 (56.05)
total	213,340 (100)	215,004 (100)	210,878 (100)	1,664 (0.78)	−2,462 (−1.15)

Changes in area are based on low and high future climate scenarios (SSP126 and SSP585) for the years 2071 to 2100. Multiple occurrences of either parental taxa or hybrids can be predicted to be present at a location and signified by ≥2 lineages. Models were projected at a 1 km^2^ scale.

### Quantifying the Ecological Impact of Mosaic Hybrid Zones through Geographic Interpolation of Suitable Landscapes for Current and Future Climates.

**Fig. 3.**
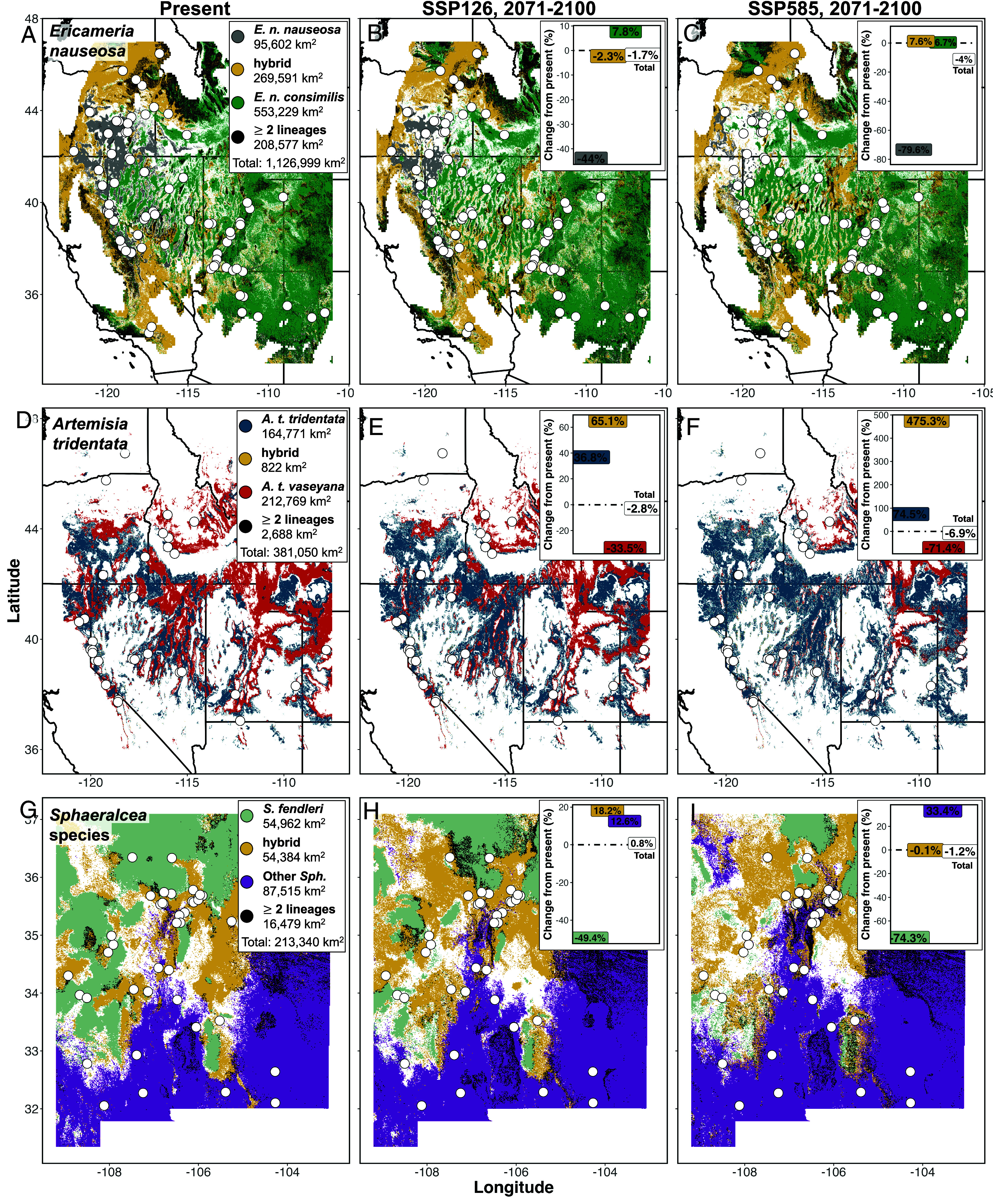
Predicted distributions of ancestry classes under present and future (SSP126 [low] & SSP585 [high], 2071 to 2100) climate scenarios. (*A*–*C*) *E. nauseosa,* (*D*–*F*) *A. tridentata*, and (*G*–*I*) *Sphaeralcea*. *Inset* legends in *A*, *D*, and *G* illustrate area for each parental taxon, hybrid, and where ≥2 lineages are predicted in the same location. *Inset* figures in *B*, *C*, *E*, *F*, *H*, and *I* show the percent change compared to the present. *E. nauseosa* and *A. tridentata* were cropped by estimated range distribution. *Sphaeralcea* was restricted to New Mexico, USA. Colors match the lineage designations in Fig. 1 and points represent sampling locations.

Given the predictability of environmentally mediated ancestry across the landscape (Fig. 2), we used the Random Forest models introduced above to interpolate the distributions of parental taxa and hybrids (Fig. 3). Consideration of hybrid ancestries expanded estimated distributions by 269,591 km^2^, 822 km^2^, and 54,384 km^2^ for *E. nauseosa*, *A. tridentata,* and *Sphaeralcea*, respectively. Differences across these estimates may in part be explained by the habitat occupied by hybrids. For example, parental *A. tridentata* taxa occupy most geographic space, whereas *A. tridentata* hybrids are constrained to a narrow geographic distribution in intermediate environments on mountain slopes, which occurs repeatedly throughout the basin and range topography of western North America (36). This habitat is difficult to accurately estimate based on the size of raster pixels representing environmental data (1 km^2^), and thus we have likely underestimated hybrid occupancy. In contrast, the proportion of environmental space occupied by hybrids compared to parents is greater in *Sphaeralcea*, which is not confined to steep slopes in montane environments (Fig. 3). We then considered low and high emission scenarios to interpolate ancestry distribution for climate change in 2071 to 2100 (SSP126 and SSP585), with results suggesting that the area occupied by hybrids will remain approximately stable or increase in each study system (Table 1). In contrast, suitable area is predicted to decrease dramatically for the parental taxa occupying environmentally cooler/wetter habitat (Fig. 3 and Table 1).

### Mosaic Hybridization Merits Ongoing Investigation.

Hybridization dynamics within our study systems warrant further investigation to understand their full scope of impacts on ecological and evolutionary processes. Given that we use ancestry class (i.e., parent or hybrid) associated with predictable environmental variation and that our sampling for each study system is not range wide, our interpolations detail geographic areas with predictable ancestry and should not be interpreted as species distribution models. We recognize additional sampling across the distributions of study systems will improve predictions; herein, our goal is to highlight potential evolutionary and ecological impacts of mosaic hybridization. Moreover, we did not include all codistributed *Sphaeralcea* species known to hybridize with *S. fendleri* (35), suggesting that broader taxonomic sampling would add complexity to ancestry predictions. However, within our sampling scheme, we note that we collected populations having morphological characteristics of the parental taxa we included. Further methodological development will allow us to predict specific hybrid ancestries across space, instead of *S. fendleri* and “Other *Sphaeralcea* species.” Finally, we excluded *A. t*. *wyomingensis*, an ecologically important tetraploid taxon within *A. tridentata. A. tridentata* subspecies are ecologically, cytologically, and chemically distinct (37–39) and despite opportunities for one-way gene flow from diploid subspecies into *A. t*. *wyomingensis* via unreduced gametes (40), a triploid block (41) prohibits opportunities for mosaic hybridization. However, we note that there are many interesting dynamics among *A. tridentata* ploidal levels, including autopolyploidization and allopolyploidization involving *A. t. tridentata* and *A. t. vaseyana*, which may break down ecological barriers and facilitate tetraploid hybridization with *A. t. wyomingensis* (40, 42). While a complete picture of the environmental distribution and ecological importance of *A. tridentata* must include *A. t*. *wyomingensis*, our goal is to highlight the ramifications of mosaic hybridization. See *SI Appendix, *Supplemental Text** for caveats and limitations to our analyses as well as confirmatory rarefaction analyses.

## Discussion

With varied impacts of hybrids on evolutionary outcomes, it stands to reason the impact of mosaic hybrid zones on ecological processes may be similarly diverse (43). For example, hybrids may act as conduits of adaptive genetic variation between parental taxa (9, 10), or they may result in loss of biodiversity by eroding species boundaries (44). Ecologically, hybrids may suture fragmented landscapes, thereby creating communities that are more resilient to disturbances (45), they may act as reservoirs of novel genetic variation that aid population persistence (11), or they may lead to population decline (44). Increasingly disturbed and fragmented landscapes combined with human-induced climate change foreshadow the importance of landscape genomic investigations to illuminate the role of mosaic hybrid zones in the persistence and continuity of plant communities. In a management context, understanding the distribution of hybrids and their environmental preferences could forestall genotype–environment mismatches, which can lead to expensive restoration failures, and incorporating hybrids into modeling efforts for predicting responses to climate change may add options for mitigation of plant community degradation.

### Linking Hybridization and Restoration Strategies to Address Habitat Fragmentation.

Ecosystem health is declining worldwide, necessitating widescale landscape management to counteract disturbances and build resilience to future change (46). In particular, habitat fragmentation damages ecosystems by hindering gene flow among species’ populations, reducing population sizes, and limiting the environmental heterogeneity to which populations are exposed (47). As a result, populations may lose genetic diversity needed for responding to selection (48). Management efforts to counteract habitat fragmentation often focus on reestablishing native plants according to the paradigm of ecologically and genetically appropriate restoration (49), regardless of whether goals support the conservation of threatened and endangered species (50) or bolster healthy community dynamics and ecosystem functions (51). This paradigm requires that native plant materials (which usually refers to seeds; 52) are matched to restoration sites by accounting for local adaptation (29, 53, 54) and evolutionary history (55). However, the basic unit of seed-based restoration is seed representing a taxon, not seed representing hybridizing taxa—even intraspecific mixing of individuals (i.e., seed and naturally occurring conspecifics at the restoration site) that are too divergent is viewed as a potentially deleterious management effort (56). Our identification of hybrids adapted to unique environments necessitates a reevaluation of using hybrid genotypes within restoration activities, as they reduce fragmentation and expand geographic footprints of ecologically important species by ~800 to 270,000 km^2^ (Table 1). Moreover, hybrids are estimated to remain important in future climates, as the environmental space they occupy is projected to expand across study systems’ geographic extents (Fig. 3).

Mosaic hybridization has consequences for seed-based restoration that depends upon the agricultural production of seed to obtain volumes necessary for broadscale restoration (57). Across our study systems, mosaic hybrid zones are facilitated by the geographic co-occurrence of parents that freely cross-pollinate despite being adapted to different environmental niches (Fig. 1). We demonstrate that hybridization produces individuals that represent a variety of ancestry combinations, and that only hybrid genotypes persist in environmental space unique and predominately intermediate from that of parental taxa (58). If environment dictates the fitness of genotypes (as in Fig. 1), then establishing an agricultural field from wild-collected, hybridized seed will select for genotypes favored by the field’s environment and result in strong genetic drift. Moreover, seed produced in the agricultural field will result from recombination and cross-pollination among plants. As such, the environments to which farm-produced seed is adapted will be unknown, and using seed in genetically appropriate restoration (i.e., matching locally adapted seed to the correct environment) may be stymied (54). However, the environmental breadth over which hybrid-origin, agriculturally produced seed can be used may also be much broader (11), or the seed may be better suited to unpredictable weather or changing climate trajectories (similar to a composite or climate-adjusted provenancing strategy in ref. 59). Ongoing management challenges that include increasingly fragmented landscapes and changing climates may require novel approaches, including using seed produced from actively hybridizing species (60).

### Mosaic Hybridization and the Persistence of Evolutionary Lineages.

Plant species that survived Pleistocene climate changes repeatedly shifted their distributions over evolutionary timescales (61, 62). Moreover, many plants that shifted their distributions are known to hybridize (63–65). The concordance of changing climates, distributional shifts, and hybridization in foundational plant clades raises the intriguing possibility that mosaic hybridization may confer evolutionary advantages to lineages. Mosaic hybridization may contribute to the persistence of parental taxa by promoting unique combinations of genetic variation (66), fostering greater community/species resilience by reducing fragmentation (47), and/or facilitating introgression (67–70). Our results demonstrate how hybrids may expand geographic ranges under different climate scenarios, although to varying degrees depending on taxa’s environmental niches (Fig. 3 and Table 1). For example, hybrids occupy 8 to 475% more geographic area compared to present in future climate scenarios, despite overall declines in the total area occupied by study systems (Table 1). While the benefits provided by mosaic hybridization to the survival of clades are speculative, understanding their diversity and scope may be critical to help taxa navigate warming climates in the increasingly fragmented landscapes of the Anthropocene.

## Materials and Methods

### Reduced Representation Sequencing, Bioinformatic Processing, and Genotype Likelihood Estimation.

Reduced representation libraries for each system were prepared using a double-digest restriction-site associated DNA sequencing (i.e., ddRADseq) method (71, 72). Genetic data for *E. nauseosa* was generated by ref. 32 and full descriptions of methods can be found therein. Similar Illumina sequencing protocols were followed for all three study systems (*SI Appendix,* Table S3). Bioinformatic processing for *E. nauseosa* and *A. tridentata* followed a custom pipeline, while stacks v2.6 was used for *Sphaeralcea*. *A. tridentata* was the only study system for which a reference genome was available (~4.2 Gb genome, nine chromosomes; 73, 74); *E. nauseosa* was aligned to a de novo reference assembly and *Sphaeralcea* was processed in stacks v2.6. Filtering was completed for all study systems using vcftools v0.1.16 (75) under similar thresholds for missing data, quality, minor allele frequency, read depth, and observed heterozygosity (*SI Appendix*, Table S3). Study systems and sampling design are discussed in detail in *SI Appendix*, Supplemental Text**. Final sample sizes after filtering for each species and ancestral classes are presented in Fig. 1 and *SI Appendix*, Table S3.

### Spatial Genetic Structure and Lineage Differentiation.

To incorporate genotype uncertainty throughout our analyses, we utilized a hierarchical Bayesian model (entropy v2.0; 76, 77) to estimate genotype probabilities for each individual at each locus (i.e., single nucleotide polymorphism), infer the number of ancestral genetic clusters (*k*), and estimate individual ancestry coefficients (*q*). Genotype probabilities from the top five models based on deviance information criterion (DIC; *k* = 2 to 9) were averaged across four chains and used for all subsequent population genetic analyses (*SI Appendix,* Table S4). Across all three study systems, a *k* = 2 model was used to delineate ancestral classes (i.e., parental or hybrid) and assign individuals by thresholds specific to each taxon (Fig. 1). Furthermore, we conducted principal component analyses (PCA) on genotype probabilities, following standardization (78), as complementary model-free approach to quantify genetic variation among all individuals (*SI Appendix,* Figs. S1 and S2). Finally, as a metric of genetic differentiation among the lineages, we estimated pairwise Hudson’s *F*_ST_ based on allele frequencies (79).

To verify that ordination and ancestry analyses were not biased by uneven sampling (80–82), we filtered variants, inferred ancestry coefficients, and conducted population genetic analyses as described below on a reduced dataset with even sampling across sampling locations and ancestry classes (*SI Appendix*, Supplemental Text**). Because patterns of clustering and ancestral class assignment were qualitatively similar for both datasets (*SI Appendix,* Fig. S8), we used the full dataset for analyses detailed below.

### Environmental Data.

We extracted environmental data across western North America for sampling sites for each study systems using the 30 climate variables of the Climatic Water Deficit Toolbox (83, 84). This dataset provides estimates of potential evapotranspiration, actual evapotranspiration, and climatic water deficit estimated from 30-y climate normals (ClimateNA 1991 to 2020, 1 km^2^ resolution; 85) and predicts spatial and distributional variation across plant communities (86, 87). Variables were reduced for each study system using variable inflation factors (VIF < 10) to account for multicollinearity. Full descriptions of these variables and their values for each sample location can be found in Dataset S1.

### Mosaicism and the Environmental Predictors of Hybridization.

As a mosaic hybrid zone is defined by hybrids occupying multiple, independent locations, thereby creating complex mosaics in which populations of parents are interspersed among populations of hybrids, we predicted that genetic variance across the parentals and the hybrids could be explained by variation in environment instead of geography. To test this, we first estimated the individual and combined influences of environment (all selected variables) and geography (latitude/longitude) on ancestry coefficients (*q*) using a variance partitioning approach (88). Next, we examined the extent to which environmental variation predicts the spatial distribution of each parental and hybrid ancestry using 1) Redundancy analysis (RDA) following the guidelines in ref. 89, a multivariate approach that quantifies the association of environmental variables and genotype probabilities, 2) univariate models of each geographic and environmental variable associated with ancestry (*q*) and ancestry class (i.e., parental or hybrid), and 3) Random Forest models of ancestry class predicted by solely environmental variation. Variance partitioning and RDA were performed using the functions *varpart()* and *rda()* in the vegan package. Univariate models were assessed using beta regressions (betareg; 90) for predictors of *q*, as *q* is bound by 0 and 1, and ANOVA (type III) in the car package (91) for associations to ancestry class. The Random Forest models were analyzed using the R package random forest (92); models were trained using 70% of the data, and accuracy was assessed using *r*^2^ and percent accurate assignment to each ancestral classification (permuted *n* = 500). Permutation tests support results of these predictive models by affirming patterns are outside what is likely due to chance (*P* < 0.001 for all study systems; *SI Appendix,* Fig. S7).

Because hybrids in our study systems occurred across the ranges of parental taxa in predictable, intermediate environmental space (Fig. 2), we projected ancestry class (i.e., parental taxa and hybrids) across geography, similar to generating genetically informed species distribution models (93). We used Random Forest to build predictive models of the occurrence of each ancestry class. Specifically, a presence/absence (0/1) model was built (trained and tested, 70/30) using the environmental data associated with sampling locations as the response variable in the R package random forest (ntrees = 1,000, mtry = default). In other words, for each model, one ancestry class was designated present (1), while the other ancestry classes represented known absences (0). The independent models were predicted across the entire estimated range of the study system [using (32) for *Ericameria,* (40) for *Artemisia*, and the New Mexico state boundary for *Sphaeralcea*], then constrained to the approximate geographic extent of our sampling. Models were combined into one prediction, with raster pixels categorized as parental, hybrids, no occurrence, or multiple occurrences (≥2 lineages present). Furthermore, we estimated how ancestry classes may shift under future climate change scenarios by utilizing the Random Forest models in association with two projected climate change scenarios (low, SSP126 and high, SSP585; 2071 to 2100). Climate change scenarios were an ensemble of eight models downscaled to a 1 km^2^ resolution (94). To highlight the ecological relevance of the hybrids across current and future climate scenarios, we calculated the extent of the area occupied by each lineage (in km^2^).

## Supplementary Material

Appendix 01 (PDF)

Dataset S01 (XLSX)

## Data Availability

Genomic data for *Ericameria, Artemisia*, and *Sphaeralcea* are available in Dryad (10.5061/dryad.1g1jwsv6r) (95) and ScienceBase (10.5066/P13M7W4G) (96).
