# Supplementary material for: Suturing fragmented landscapes: Mosaic hybrid zones in plants may facilitate ecosystem resiliency
Source: Proc Natl Acad Sci U S A. 2025 Jul 28;122(31):e2410941122. doi: 10.1073/pnas.2410941122 (PMC12337288; doi:10.1073/pnas.2410941122)
Supplement: Supplementary file 1 — Appendix 01 (PDF) [file pnas.2410941122.sapp.pdf]

PNAS

Supporting Information for

Suturing fragmented landscapes: Mosaic hybrid zones in plants may facilitate ecosystem resiliency

Rob Massatti*, Trevor M. Faske*, Ivana M. Barnes, Elizabeth A. Leger, Thomas L. Parchman, Bryce A. Richardson, L. Lacey Knowles

Rob Massatti
Email: rmassatti@usgs.gov

This PDF file includes:

Supporting text
Figures S1 to S9
Tables S1 to S4
Legend for Dataset
SI References

Other supporting materials for this manuscript include the following:

Dataset

Supporting Information Text

Study system and sampling design. We highlight convergent hybridization patterns across three widespread suites of taxa critical for restoration and conservation efforts in western North America: *Ericameria nauseosa* (Pall. ex Pursh) G.L. Nesom & G.I. Baird (rubber rabbitbrush; Asteraceae), *Artemisia tridentata* Nutt. (big sagebrush; Asteraceae), and *Sphaeralcea fendleri* A. Gray (Fendler's globemallow; Malvaceae). *Ericameria nauseosa* and *A. tridentata* are foundational shrub species spanning the entirety of western North America, and *S. fendleri* is a forb commonly occupying disturbed soils across the western drylands. Within study systems, we only included individuals in datasets that passed genetic filtering, and we assigned individuals as either a parental taxon or a hybrid using ancestry coefficient (*q*) cutoffs (Fig. 1 and Dataset).

Ericameria nauseosa is a perennial shrub that flowers in the late summer, hosts a diverse array of pollinators, has substantial phenotypic and genetic variation (1–3), and has extended community and ecosystem-level consequences (4–6). *Ericameria nauseosa* consists of two recognized subspecies (*E. n. nauseosa* [grey] and *E. n. consimilis* [green]) that geographically overlap but are phenotypically distinguishable by variation in grey and green pigmentation in the leaves and stems (7). Within these subspecies, there are an additional 22 named varieties (3). Despite sympatric distributions, the subspecies form genetically distinct lineages wherein hybrids occupy environmentally predictable habitat (2). We leverage data from Faske *et al.,* (2), which includes a total of 586 individuals that passed genetic filtering spanning both subspecies and 14 of the varieties. Specifically, we include *E. n. nauseosa,* consisting of 276 individuals across 27 populations, *E. n. consimilis,* consisting of 185 individuals across 35 populations, and hybrids, consisting of 125 individuals across 28 populations.

Artemisia tridentata is among the most ecologically significant plants of western North America, where it dominates arid shrublands from northern Mexico to southern Canada. The *A. tridentata* complex consists of three subspecies: basin (*A. t. tridentata*), Wyoming (*A. t. wyomingensis*), and mountain (*A. t. vaseyana*), which have both diploid and tetraploid forms and are associated with specific ecological niches within overlapping geographic ranges. Here, we focus on just the diploid populations of *A. t. tridentata* and *A. t. vaseyana*, as hybridization across diploids and tetraploids is unlikely (but see [8]) and gene flow that occurs into *A. t. wyomingensis* does not meet the criteria of mosaic hybridization that we discuss herein. Diploid subspecies within *A. tridentata* are evolutionarily distinct, but form hybrids (9, 10), and our sampling includes 201 individuals across 16 populations for *A. t.* ssp. *tridentata,* 158 individuals across 16 populations for *A. t. vaseyana*, and 38 individuals across ten populations for hybrids.

Sphaeralcea is a widespread perennial forb found across western North America, containing approximately 25 species that hybridize frequently (11). Due to the importance of *Sphaeralcea* to pollinators, for foraging for a wide range of small and large animals, and in establishing in harsh growing conditions and after disturbances, they are highly valued in restoration and rehabilitation applications and seeds are actively collected by the U.S. Department of Interior Bureau of Land Management Seeds of Success program. Herein, we focus on *S. fendleri* (25 pop., 247 indv), but we include individuals identified as *S. incana* (7 pop., 39 indv), *S. hastulata* (5 pop., 30 indv), *S. angustifolia* (6 pop., 45 indv.), and *S. parvifolia* (2 pop., 6 indv); many of these individuals were morphologically intermediate and field identifications were tentative. Leaf tissues were collected by the New Mexico Bureau of Land Management, in conjunction with the Institute for Applied Ecology Southwest Seed Partnership (https://southwestseedpartnership.org/), to promote native plant materials development.

Reduced representation sequencing, bioinformatic processing, and filtering. All sequencing information, bioinformatic processing, and filtering across the study systems are represented in Table S3. We developed the *E. nauseosa* and *A. tridentata* sequencing libraries using the same protocol; library development for *Sphaeralcea* was slightly different, as noted below. For each study system, dried leaf material from each individual was ground into powder using a Qiagen TissueLyser and DNA was extracted using Qiagen DNeasy Plant kits (Qiagen, Valencia CA). Reduced representation libraries were prepared using a double-digest restriction-site associated DNA sequencing (ddRADseq) method (12, 13). We used the restriction endonucleases *Eco*RI and *Mse*I to digest genomic DNA (*Eco*RI and *Msp*I for *Sphaeralcea*), after which we ligated unique barcodes and Illumina adaptors using T4 DNA ligase (New England Biolabs, Ipswich MA). Barcoded fragments were PCR amplified with Iproof DNA polymerase (BioRad, Hercules CA), and fragments ranging from 350 to 450 bp (400 to 600 bp for *Sphaeralcea*) were size selected using a Pippin Prep quantitative electrophoresis unit (Sage Science, Inc). Single-end sequencing (100 bp read lengths) was performed using the Illumina HiSeq 4000 platform at the University

of Wisconsin for *E. nauseosa*, Illumina NovaSeq 6000 at the University of Texas Genomic Sequencing and Analysis Facility (UT GSAF), and Illumina NovaSeq 6000 at University of Oregon for *Sphaeralcea* species.

For *E. nauseosa* and *A. tridentata,* we detected and discarded sequences representing potential contaminants (*E. coli* and PhiX) or various Illumina-associated oligos using a pipeline of Perl and bash scripts (http://github.com/ncgr/tapioca). We used a custom Perl script to correct barcode sequencing errors, trim cut site and barcoded oligo-associated bases, and demultiplex reads by individual. As a reference genome is not available for *E. nauseosa* or a close relative, we used a *de novo* assembly of unique reads to create a consensus reference of genomic regions sampled by our reduced representation approach (reference assembly, hereafter). We optimized parameters for this step optimized using shell scripts and documentation provided for dDocent (14; cutoffs: individual = 8, coverage = 5; clustering similarity: -c .92), and the reference assembly was produced using CD-HIT-EST V4.8.1 (15). For *A. tridentata,* we used a haploid pseudo-chromosome genome of *A. t. tridentata* for alignment across a ~4.2 Gb genome with 9 chromosomes (16, 17). We mapped demultiplexed reads for each individual to the reference assembly using BWA-MEM V0.7.17 (18) and identified sequence variants using BCFTOOLS V1.9 (19).

For Sphaeralcea, raw data were demultiplexed and cleaned (i.e., to exclude raw reads containing more than four low-quality sites and/or adapter contamination) using custom scripts, *fastq-multx* in ea-utils (20), and *process_radtags* in STACKS V2.6 (21). The remaining processing was accomplished using STACKS and followed the *r80* protocol detailed in Rochette and Catchen (22). Parameters affecting the assembly were assessed based on how parameter combinations affected *r80* loci (i.e., those found in 80% of samples or more); the optimal parameter set associated with the plateau of the number of *r80* loci was selected (22, 23). Values used in the final assembly were: minimum depth of coverage to initiate a new stack (*-m* in *ustacks*) = 3; the number of mismatches allowed between stacks (*-M* in *ustacks*) = 7; distance allowed between catalog loci (*-n* in 3 *cstacks*) = 7. The *populations* program in stacks was run to generate the dataset used in subsequent filtering (see below) and as such did not include filtering parameters.

Filtering parameters vary slightly for each study system and ranges will be provided in the text below (see Table S3 for exact parameters). Datasets were filtered for all study systems using VCFTOOLS V0.1.16 (24). We retained only biallelic loci covered by reads present in at least 60 – 70% of individuals, thinned to one locus per 100 bp (one locus per contig for *E. nauseosa* and *Sphaeralcea* sp.) to reduce effects of linkage disequilibrium and sequencing error. In addition, individuals missing data for greater than 40 – 50% of loci were removed before further analyses. After additional filtering with custom Python scripts, we retained loci with minor allele frequency (MAF) ≥ 0.01 – 0.02, mean read depth across individuals, and alternate allele call quality. Filtering cutoffs were decided based on distributions of the metrics in question, with the goal of retaining the highest quality loci without sacrificing genomic coverage. We removed loci with excessive read depth and only retained loci with two alleles present to ameliorate genotyping bias from the potential mis-assembly of paralogous genomic regions (25, 26). Lastly, we retained loci with F_{IS} < –0.5, as mis-assembly of paralogous genomic regions can lead to abnormal heterozygosity (27, 28).

Estimating ancestry with ENTROPY. To estimate genotype probabilities for each individual at each locus, infer the number of ancestral genetic clusters (*k*), and estimate individual ancestry coefficients (*q*), we utilized a hierarchical Bayesian model that incorporates genotype uncertainty (ENTROPY V2.0; 29, 30). entropy utilizes an allele frequency prior and incorporates genotype uncertainty arising from sequencing and alignment error, as well as stochastic variation among individuals and loci in sequencing depth during parameter estimation (29). Using genotype likelihoods calculated from BCFTOOLS, we executed *k*-means clustering of the first five PCs to generate starting values of ancestry coefficients (*q*) to seed the MCMC (29). ENTROPY was used to estimate genotype probabilities and ancestry coefficients for models based on a range of ancestral genetic clusters (*k*). We ran 60,000 MCMC iterations across 4 chains with a burn-in of 10,000, thinning every tenth step, for models based on each level of *k*. Genotype probabilities from the top five models based on DIC (*k* = 2 – 9) were averaged across all chains and used for all subsequent population genetic analyses (Table S4).

Estimating genetic diversity. We estimated nucleotide diversity (θ_{π},; Fig. S9 [31]) and the Watterson estimator (θ_W; [32]) within each population (min. 3 individuals) using methods that incorporate genotype

uncertainty implemented in ANGSD V 0.923 (33, 34). Using individual bam files, we estimated the folded site allele frequency likelihoods with "realsfs" and calculated genotype likelihoods using the setting "GL 1" estimated from the model implemented in SAMTOOLS model to obtain the likelihood of the folded site frequency spectrum (SFS). We estimated diversity (θ_π and θ_W) and Tajima's *D* (35) using "doThetas 1" and "thetastat" commands using SFS likelihoods as priors, respectively, for each locus across the reference assembly and averaged the measures for each population.

Rarefaction of genomic datasets. As it is widely known that uneven sampling in ordination and clustering analyses can bias patterns of differentiation in predictable ways (36–38), we created rarefied dataset to verify that ordination and ancestry analyses were not biased by uneven sampling. Each taxon was down sampled to have similar numbers of individuals and sampling locations within each lineage, as well as similar numbers of individuals within a sampling location. We re-filtered variants, inferred ancestry coefficients, and conducted population genetic analyses as described below on a reduced data set with even sampling across sampling locations and study systems. Patterns of clustering and ancestral class assignment were qualitatively similar for both datasets (Fig. S8).

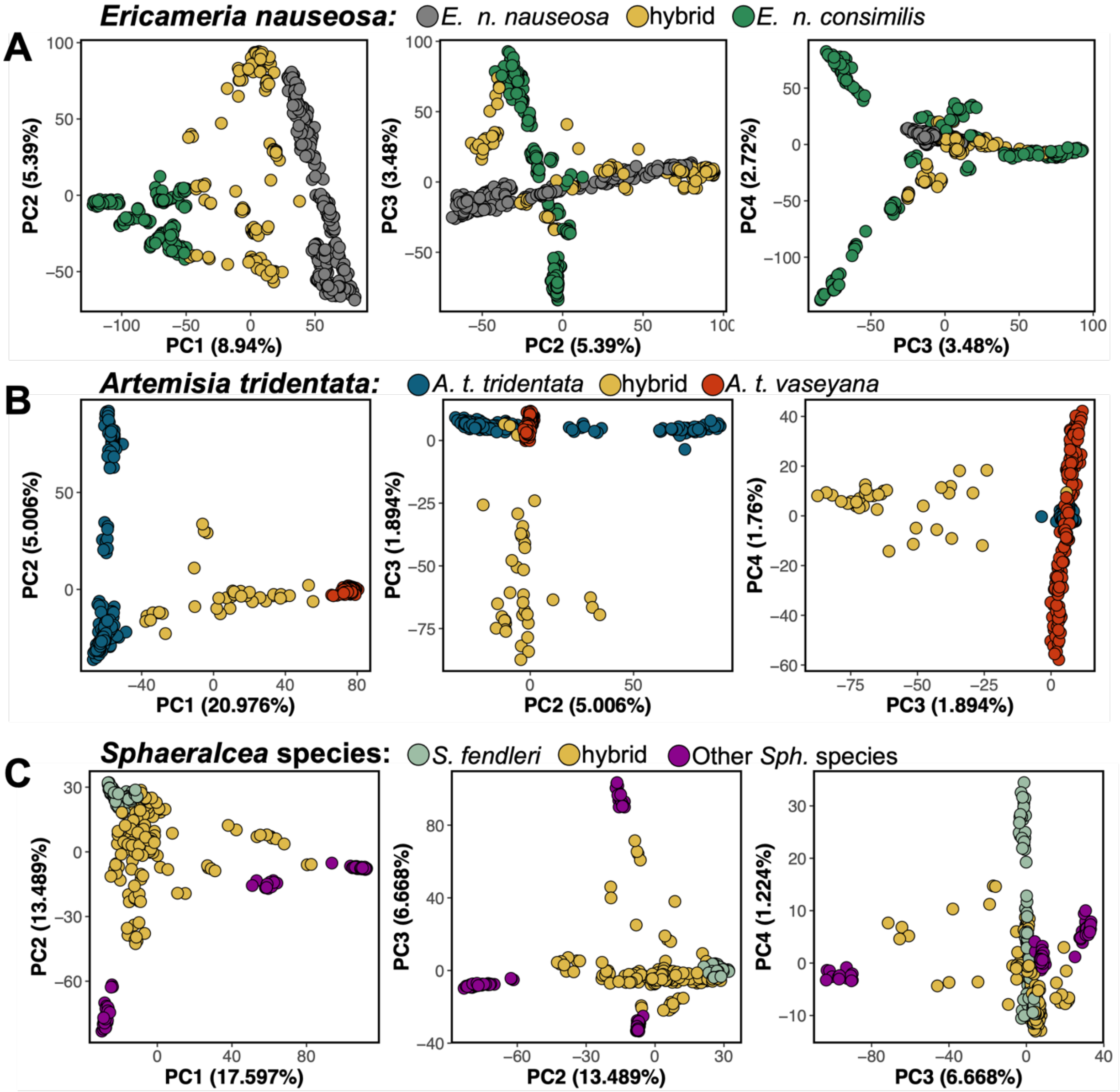

Figure S1. Principal components analysis (PCA, axes 1 – 4) of genotype probabilities for (A) *Ericameria nauseosa*, (B) *Artemisia tridentata*, and (C) *Sphaeralcea* species. Percent variation explained by each axis is noted parenthetically, and hybrids are colored yellow.

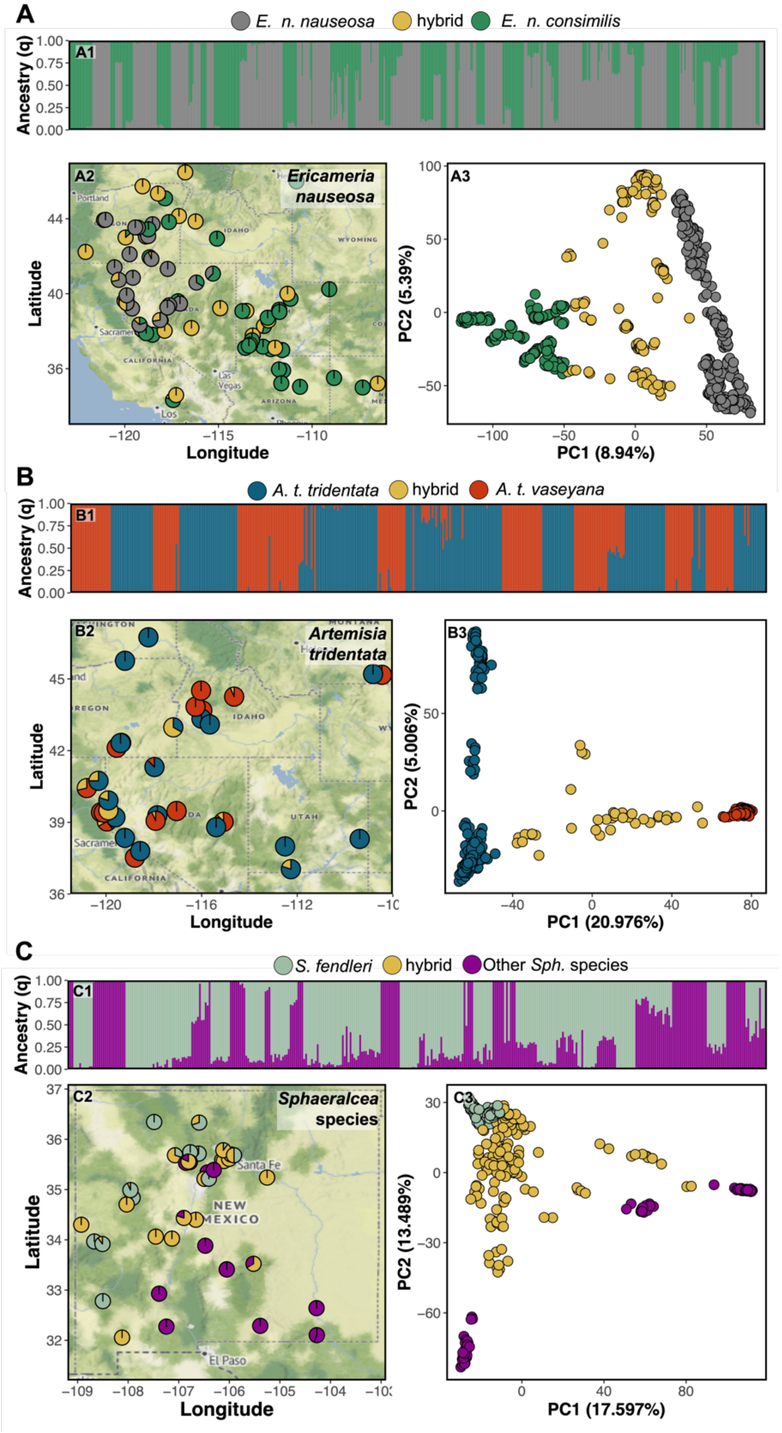

Figure S2. Across all study systems, parental taxa are independent evolutionary lineages and co-occur geographically. (**A**) *Ericameria nauseosa,* (**B**) *Artemisia tridentata*, and (**C**) *Sphaeralcea* sp. (**1**) Ancestry coefficients (*q*) for each individual from the hierarchical Bayesian model of ENTROPY for k = 2, grouped by sampling location. Color proportion of each bar is ancestry proportion. (**2**) Map illustrating sampling locations. Pie chart colors correspond to the proportion of individuals at a locality assigned to each lineage or as a hybrid. (**3**) Principal component analysis (PCA) illustrates genetic differentiation between lineages and the presence of hybrid individuals. Colors indicate parental or hybrid classification based of ancestry coefficients (see Fig. 1 for cutoffs and sample sizes).

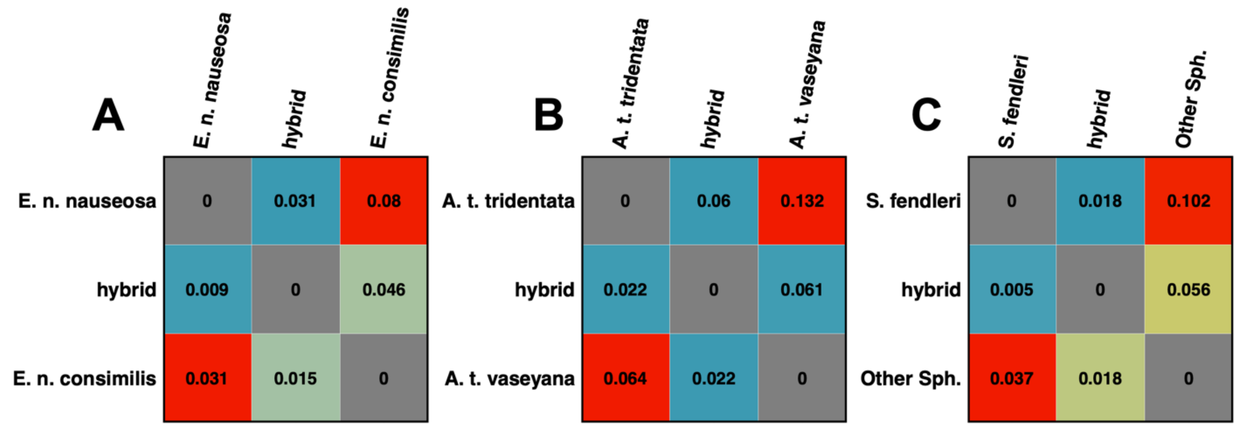

Figure S3. Pairwise estimates of genetic differentiation across parentals and hybrids for *Ericameria nauseosa* (**A**), *Artemisia tridentata* (**B**), and *Sphaeralcea* species (**C**). Hudson's F_{ST} is represented in the upper right corner and Nei's *D* is represented in the lower left corner. Colors correspond to the magnitude of estimates, with warmer colors corresponding to higher values.

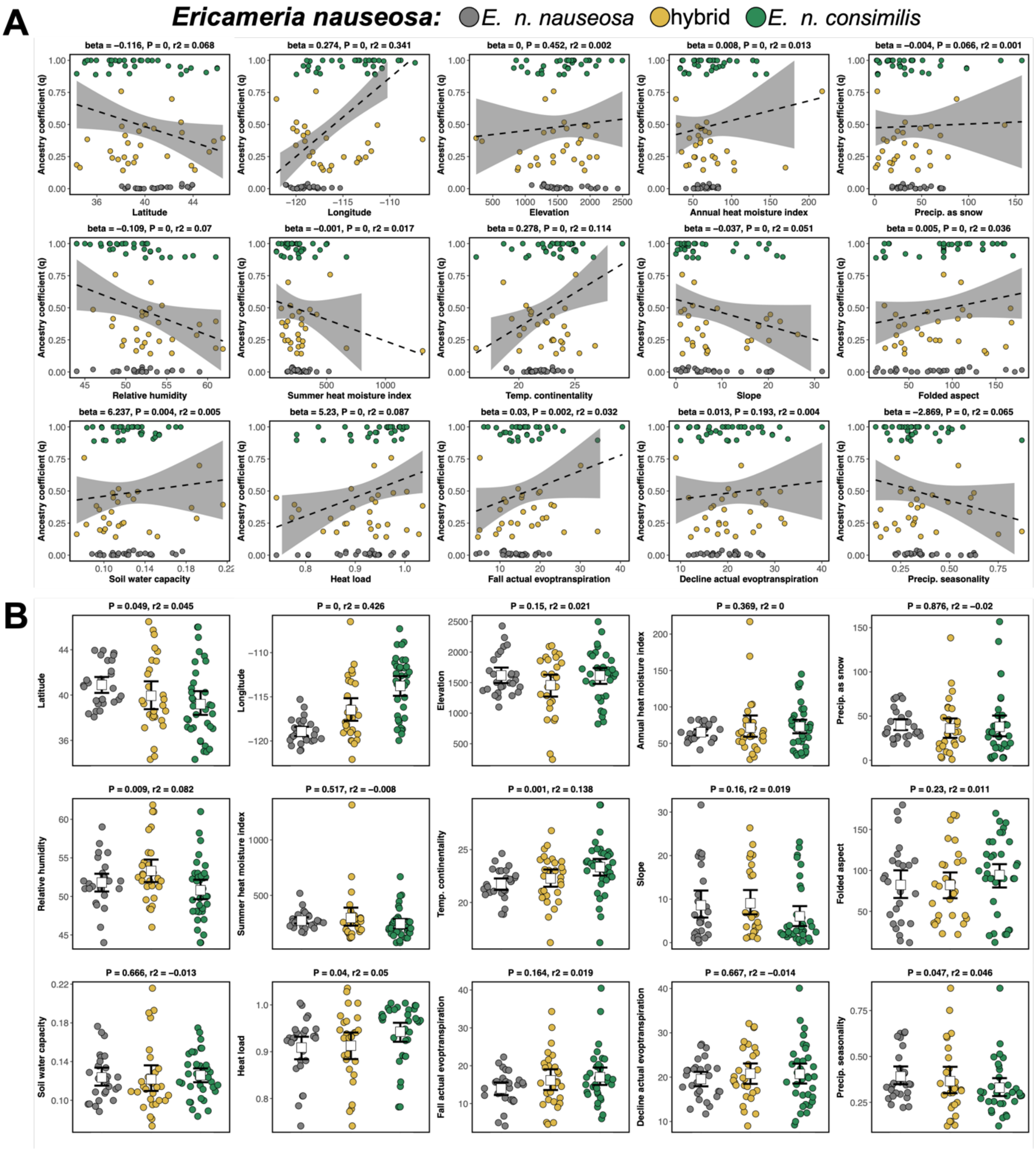

Figure S4. Geographic and environmental variation varies by ancestry and ancestral categorization for *Ericameria nauseosa*. (**A**) Univariate scatter plots of each variable to ancestry coefficients with fitted line and standard error estimated using the stat_smooth() function in GGPLOT2 in R. As ancestry coefficients are bound from 0 to 1, beta regressions were used to assess the relationship between q and predictor variables with beta coefficients, *P*-values, and r^2 reported above each panel. (**B**) Relationship between geographic or environmental variables and ancestral categorization. Circles represent the raw data colored by parentals or hybrids while white squares and error bars represent the mean and the 95% bootstrapped confidence interval. *P*-values and r^2 were assessed using univariate ANOVA (type III) and are reported above each panel. Colors correspond to ancestral classification of individuals.

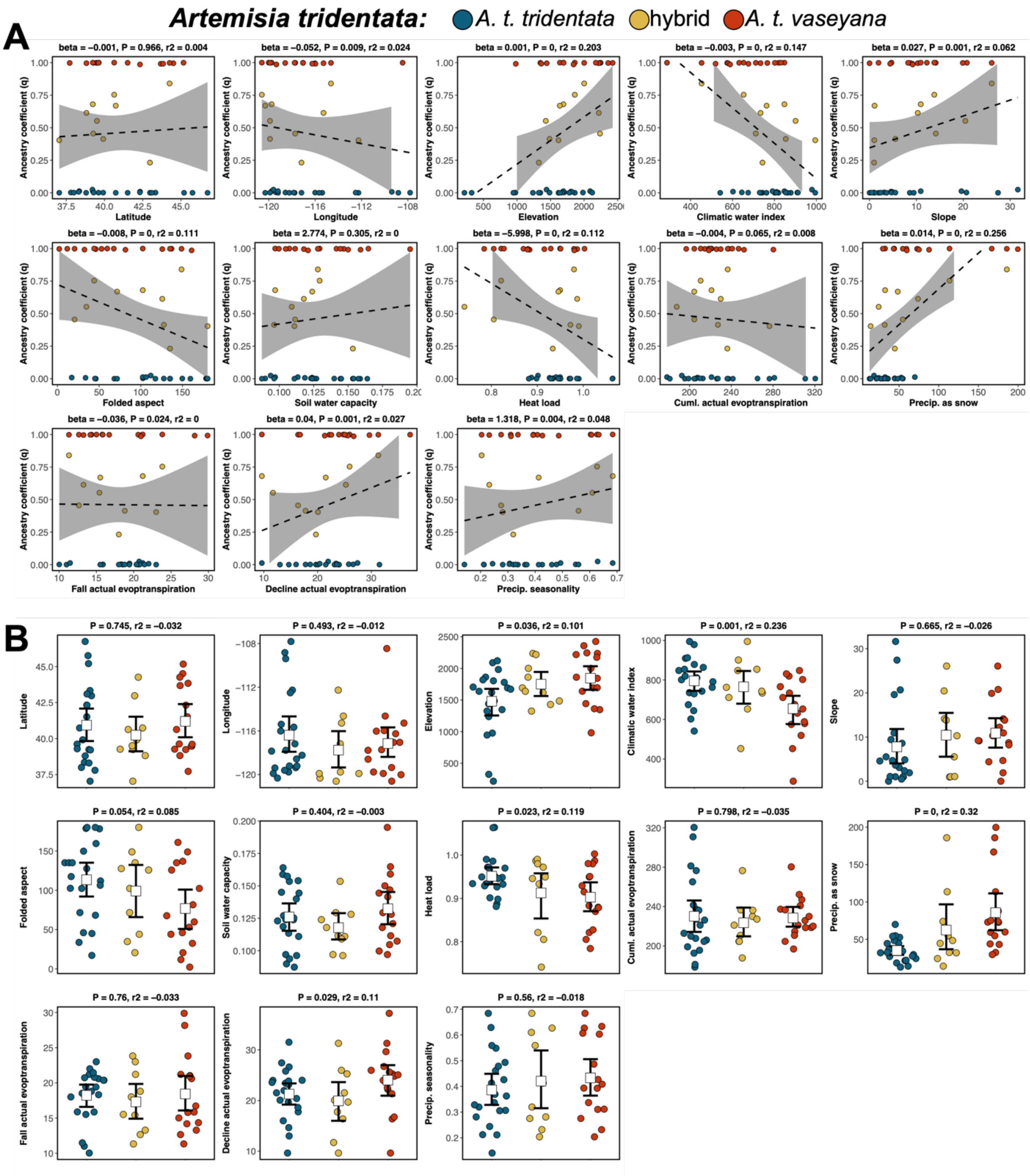

Figure S5. Geographic and environmental variation varies by ancestry and ancestral categorization for *Artemisia tridentata*. (**A**) Univariate scatter plots of each variable to ancestry coefficients with fitted line and standard error estimated using the stat_smooth() function in GGPLOT2 in R. As ancestry coefficients are bound from 0 to 1, beta regressions were used to assess the relationship between q and predictor variables with beta coefficients, *P*-values, and r^2 reported above each panel. (**B**) Relationship between geographic or environmental variables and ancestral categorization. Circles represent the raw data colored by parentals or hybrids while white squares and error bars represent the mean and the 95% bootstrapped confidence interval. *P*-values and r^2 were assessed using univariate ANOVA (type **III**) and are reported above each panel. Colors correspond to ancestral classification of individuals.

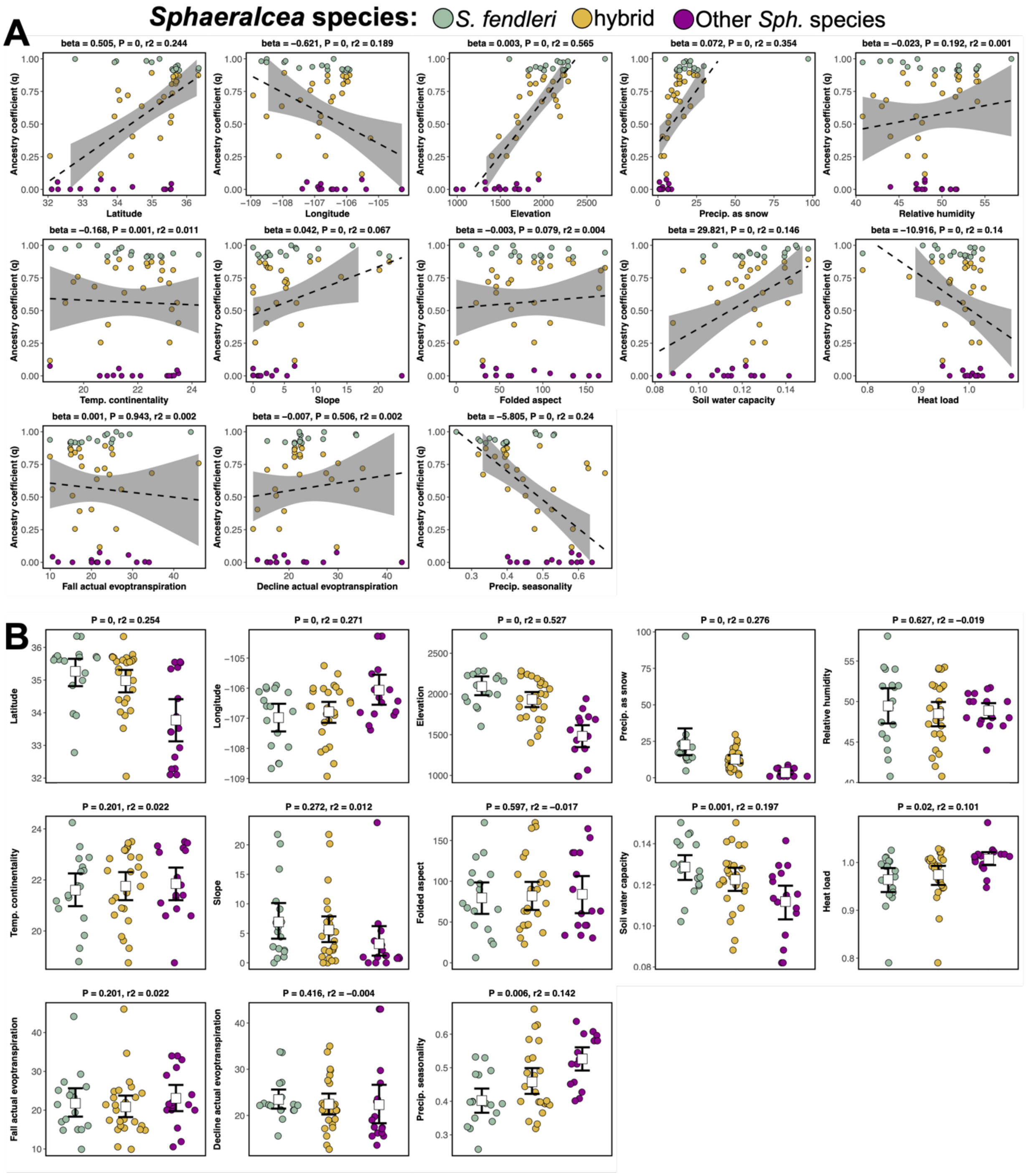

Figure S6. Geographic and environmental variation varies by ancestry and ancestral categorization for *Sphaeralcea*. (**A**) Univariate scatter plots of each variable to ancestry coefficients with fitted line and standard error estimated using the stat_smooth() function in GGPLOT2 in R. As ancestry coefficients are bound from 0 to 1, beta regressions were used to assess the relationship between q and predictor variables with beta coefficients, *P*-values, and r^2 reported above each panel. (**B**) Relationship between geographic or environmental variables and ancestral categorization. Circles represent the raw data colored by parentals or hybrids while white squares and error bars represent the mean and the 95% bootstrapped confidence interval. *P*-values and r^2 were assessed using univariate ANOVA (type III) and are reported above each panel. Colors correspond to ancestral classification of individuals.

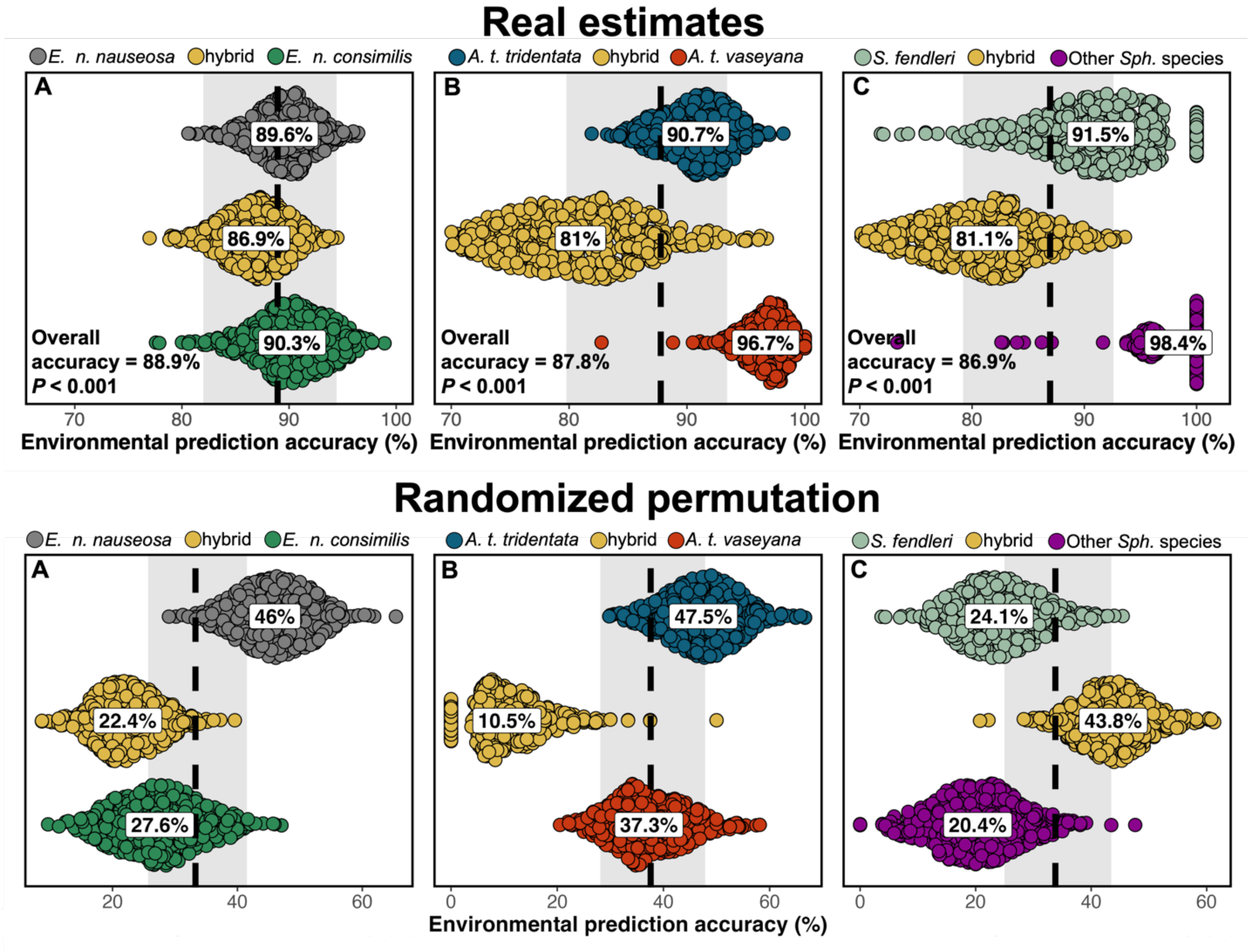

Figure S7. Permutation tests for Random Forest accuracy for environmental predictions of identity (parental vs. hybrid) using Random Forest for *Ericameria nauseosa* (**A**), *Artemisia tridentata* (**B**), and *Sphaeralcea* sp. (**C**). The *top* panel represents the real point estimates and is the same as Fig. 2 of the main text. The *bottom* panel represents the randomized permutation test values. The same models for both analyses were used but the *bottom* had randomly assigned an individual a parental or hybrid classification. Comparing the original model to the permuted model highlights that the estimated values are outside those predicted by chance alone (*P*s < 0.001). Points represent permuted accuracy within each lineage (text boxes show mean accuracy). The dashed line and shaded bar represent the overall mean predictive accuracy and 95% confidence interval.

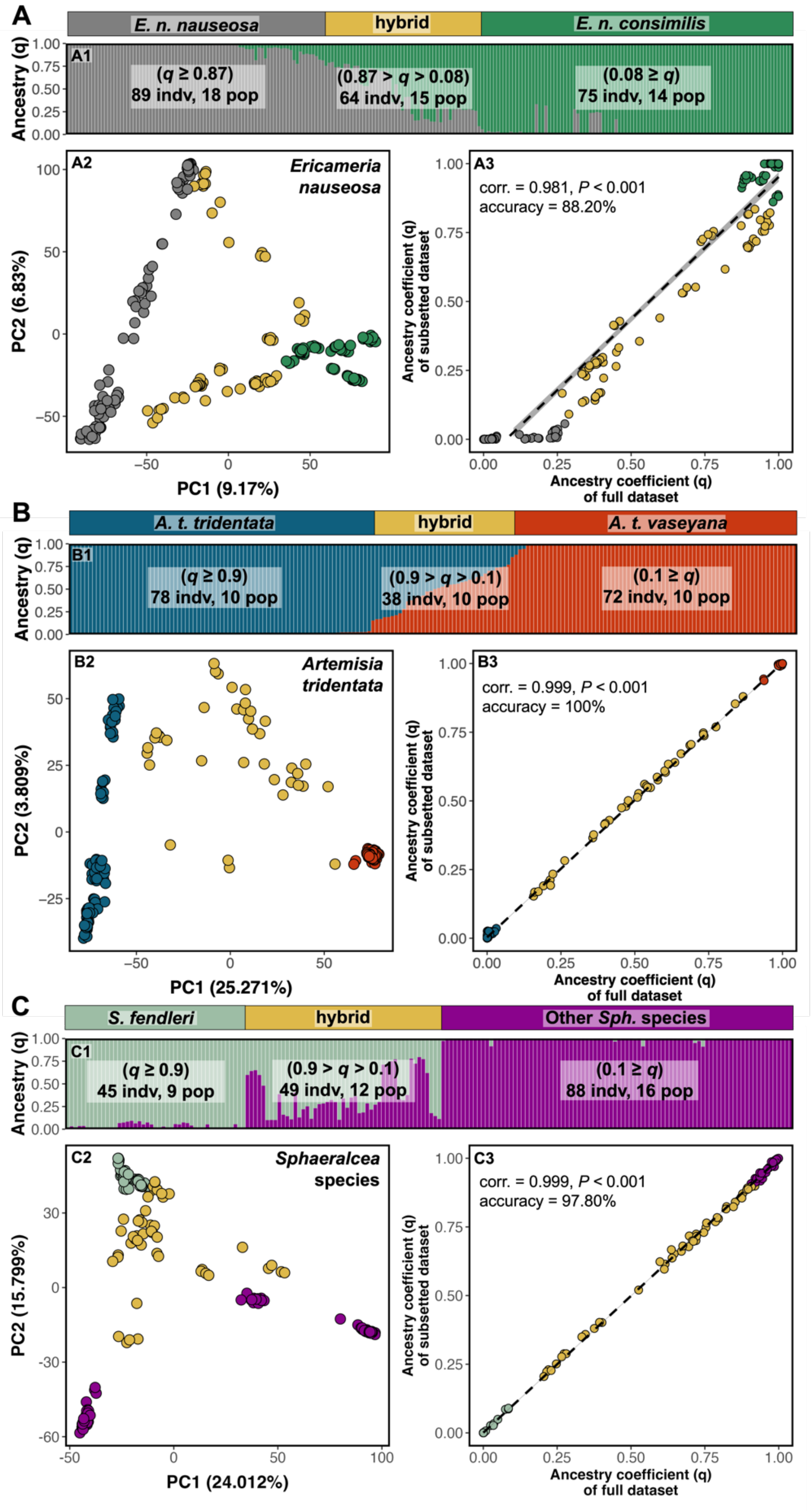

Figure S8. Recapitulations of the genetic variation across all study systems with a rarified dataset with roughly equal sample sizes across lineages and populations. (**A**) *Ericameria nauseosa,* (**B**) *Artemisia tridentata*, and (**C**) *Sphaeralcea* sp. (**1**) Ancestry coefficients (q) for each individual from the hierarchical Bayesian model of ENTROPY for k = 2, ordered by the first PCA axis. Color proportion of each bar is ancestry proportion. (**2**) Principal component analysis (PCA) illustrates genetic differentiation between lineages and the presence of hybrid individuals. Colors indicate parental or hybrid classification based of ancestry coefficients. (**3**) Relationship of $k = 2$ ancestry coefficients from ENTROPY for the full dataset and the rarified dataset. Pearson's correlation, *P*-values, and the percent match between the two datasets is reported.

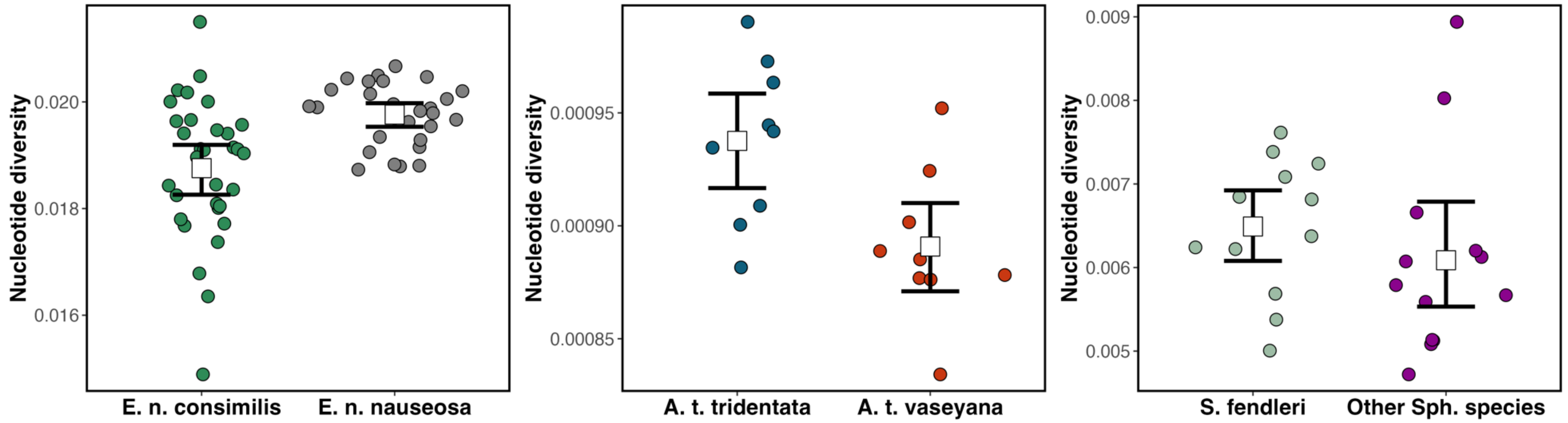

Figure S9. Nucleotide diversity (θ_{π}; 31) estimates for each parental taxa. Circles represent the raw data colored by parentals while white squares and error bars represent the mean and the 95% bootstrapped confidence interval. *E. nauseosa* and *Sphaeralcea* sp. were estimated at the population level while A. *tridentata* was estimated at the chromosome level.

Table S1. Variance partitioning of individual and shared effects of geography and environment on ancestry coefficients across each study system. Latitude and longitude were included to encapsulate geography and all environmental variables (see Table S3) made up environment. The first three lines refer to the overall contribution of those variables on ancestry while the next three refer to the individual contributions with '|' representing conditioning on that variable. Residuals refer the amount not explained by the model.

	Adjusted R^2		
**Model**	*E. nauseosa*	*A. tridentata*	*Sphaeralcea* sp.
**ancestry ~ geo + env**	0.486	0.581	0.783
**ancestry ~ geo**	0.380	0.019	0.479
**ancestry ~ env**	0.351	0. 535	0.783
**ancestry ~ geo | env**	0.135	0.046	0.000
**ancestry ~ env | geo**	0.105	0.562	0.304
**ancestry ~ combined**	0.245	0.000	0.479
**Residuals**	0.514	0.419	0.217

Table S2. Importance values for environmental predictors within study systems resulting from redundancy analysis (RDA) and Random Forest (RF). Included variables have variance inflation factors (VIF) ≤ 10.

	E. nauseosa					*A. tridentata*					*Sphaeralcea* sp.				
**Env. variable**	VIF	RDA loading	RDA rank	RF imp.	RF rank	VIF	RDA loading	RDA rank	RF imp.	RF rank	VIF	RDA loading	RDA rank	RF imp.	RF rank
Precip. as snow	4.733	0.154	9	6.00	7	6.366	0.801	1	23.39	1	4.897	0.54	2	15.48	1
Heatload	5.803	0.381	3	11.42	2	6.123	0.473	5	9.59	2	2.561	0.303	4	3.61	4
Climatic water index		–	–	–	–	6.081	0.655	2	8.67	3		–	–	–	–
Elevation	6.738	0.186	8	4.28	10	5.202	0.513	3	7.63	4	8.902	0.909	1	13.26	2
Fall actual evapotranspiration	3.103	0.264	7	4.20	11	2.680	0.144	10	6.64	5	2.816	0.142	8	2.31	7
Precip. seasonality	4.560	0.529	2	5.23	8	2.556	0.15	9	5.48	6	6.181	0.525	3	4.53	3
Decl. actual evapotranspiration	3.050	0.017	13	3.91	12	2.961	0.303	6	5.43	7	3.008	0.256	6	2.66	6
Cuml. actual evapotranspiration		–	–	–	–	6.464	0.091	11	5.16	8		–	–	–	–
Folded aspect	3.285	0.276	5	3.71	13	3.443	0.479	4	4.45	9	2.300	0.14	9	0.68	11
Slope	2.828	0.145	11	6.90	6	2.569	0.226	7	3.43	10	1.885	0.281	5	1.89	9
Soil water capacity	3.183	0.091	12	4.65	9	5.323	0.217	8	3.33	11	1.911	0.238	7	1.49	10
Relative humidity	4.311	0.306	4	9.04	4		–	–	–	–	4.152	0.077	10	2.69	5
Temp. continentality	3.248	0.649	1	18.85	1		–	–	–	–	4.979	0.033	11	1.99	8
Summer heat moisture index	6.424	0.151	10	10.88	3		–	–	–	–		–	–	–	–
Annual heat moisture index	9.159	0.269	6	8.16	5		–	–	–	–		–	–	–	–

Table S3. Sequencing and bioinformatic information and protocols for each study system.

	E. nauseosa	***A. tridentata***	***Sphaeralcea* sp.**
**Reduced representation libraries**	ddRADseq	ddRADseq	ddRADseq
**restriction enzymes**	EcoRI & MseI	EcoRI & MseI	EcoRI & MspI
**Pippin prep size selection**	350-450bp	350-450bp	400-600bp
**sequencing type**	single-end	single-end	single-end
**sequencing platform**	Illumina HiSeq 4000	Illumina NovaSeq 6000	Illumina NovaSeq 6000
**sequencing facility**	University of Wisconsin	UTGSAF	University of Oregon
**decontamination / trimming**	github.com/ncgr/tapioca	github.com/ncgr/tapioca	*process_radtags* (STACKS)
**demultiplexing**	custom Perl script	custom Perl script	*fastq-multx* (Aronesty 2011)
**avg. reads per indv.**	2276267	2276703	1872931
**reference assembly**	*de novo*	genome	*de novo*
**reference assembly method**	cd-hit-est v4.8.1	Melton *et al.*, 2022	STACKS v2.6
**alignment method**	bwa-mem v0.7.17	bwa-mem v0.7.17	STACKS v2.6
**variant calling method**	bcftools v1.9	bcftools v1.9	STACKS v2.6
**num. of indv.**	600	407	366
**num. of loci before filtering**	1613057	2766918	88140
**filtering method**	vcftools v0.1.16	vcftools v0.1.16	vcftools v0.1.16
**minor allele freq. (MAF)**	0.02	0.01	0.02
**max. missing data (loci)**	30%	40%	40%
**thinned**	100bp	100bp	100bp
**max. missing data (indv.)**	40%	50%	50%
**min. mean read depth**	3	2	10
**max. mean read depth**	25	25	50
**min. quality**	750	100	999
**min. Fis**	-0.5	-0.5	-0.5
**final mean coverage per indv.**	7.25x	3.18x	24.69x
**final num. of indv.**	586	397	366
**final num. of loci**	22917	13003	7271

Table S4. Deviance information criterion (DIC) values for different ancestral clusters (k) outputted from ENTROPY across four chains. The data best model(s) is indicated by lowest DIC value and presented in bold.

	k	chain 1	chain 2	chain 3	chain 4	mean
E. nauseosa						
	**2**	**31235761.82**	**31363445.98**	**31660942.42**	**31825995.42**	**31518422.34**
	3	33145796.79	33228694.28	33739298.47	34535151.69	33602822.33
	4	34280800.92	34847580.16	35433797.03	35667550.29	35085184.27
	5	33518588.06	33926279.57	34256212.05	34466287.03	34058309.72
	6	35658707.76	36304858.22	37514473.47	37572699.02	36811678.36
	7	36777341.52	36976124.13	38706378.32	41239748.01	38230349.07
	8	83617352.63	133895982.7	235957096.2	301829319.8	187525471.7
	9	145255251.6	350119378	648345751.7	856240278.3	499737631.6
A. tridentata						
	**2**	**15957430.44**	**15664199.85**	**15415462.89**	**16170073.44**	**15801791.66**
	3	17055204.5	16685822.4	16513412.4	16939371.46	16798452.69
	4	16864273.06	16504852.07	16525725.88	16580390.66	16618810.42
	5	19981272.16	20936337.9	19618211.98	20358179	20223500.26
	6	22670787.93	22049330.66	23521862.28	21076213.51	22329548.6
	7	25629554.66	29629899.19	24179463.63	27903547.76	26835616.31
***Sphaeralcea* sp.**						
	**2**	**11789226.1**	**12074800.1**	**11545490.8**	**11656802.2**	**11766579.8**
	3	12589627.4	12317920.6	12456998.3	12736015.6	12525140.5
	4	11102608.5	11962954	11811757.7	12332157.5	11802369.4
	5	307022193	282883071	286281350	229805744	276498090
	6	2154681205	1953525254	1870259147	2112671758	2022784341
	7	2808738753	898743836	2071765309	438700042	1554486985
	8	38671615.6	44844292.9	77921667.5	85338238.6	61693953.6
	9	910976776	310393886	395439198	686902402	575928066

Dataset (separate file). An excel file with information for each of the three taxa kept separately in a tabular form. Tabular sheets include: (1) Information on each individual sampling location with ancestral classification and ancestry coefficients, (2) Population (i.e., sampling location) level environmental data, (3) Population-level pairwise F_{ST}, and (4) Population-level diversity estimates of nucleotide diversity ($\boldsymbol{\theta_\pi}$), the Watterson estimator ($\boldsymbol{\theta_W}$), and Tajima's *D*. This file also contains the metadata for each tab within and descriptions of the variables.

SI References

1. T. M. Faske, *et al.*, Genomic and common garden approaches yield complementary results for quantifying environmental drivers of local adaptation in rubber rabbitbrush, a foundational Great Basin shrub. *Evolutionary Applications* **14**, 2881–2900 (2021).

2. T. M. Faske, *et al.*, Environment predicts the maintenance of reproductive isolation in a mosaic hybrid zone of rubber rabbitbrush. *Evolution* **78**, 300–314 (2024).

3. G. L. Nesom, G. I. Baird, Completion of Ericameria (Asteraceae: astereae), diminution of Chrysothamnus. *Phytologia* **75**, 74_93.

4. K. D. Floate, G. W. Fernandes, J. A. Nilsson, Distinguishing intrapopulational categories of plants by their insect faunas: galls on rabbitbrush. *Oecologia* **105**, 221–229 (1996).

5. E. D. McArthur, C. F. Tiernan, B. L. Welch, Subspecies specificity of gall forms on Chrysothamnus nauseosus. *Great Basin Naturalist* **39**, 81–87 (1979).

6. D. F. Hegerhorst, D. J. Weber, E. D. McArthur, A. J. Khan, Chemical analysis and comparison of subspecies of Chrysothamnus nauseosus and other related species. *Biochemical Systematics and Ecology* **15**, 201–208 (1987).

7. L. C. Anderson, THE CHRYSOTHAMNUS-ERICAMERIA CONNECTION (ASTERACEAE).

8. P. Monnahan, *et al.*, Pervasive population genomic consequences of genome duplication in Arabidopsis arenosa. *Nat Ecol Evol* **3**, 457–468 (2019).

9. B. A. Richardson, J. T. Page, P. Bajgain, S. C. Sanderson, J. A. Udall, Deep sequencing of amplicons reveals widespread intraspecific hybridization and multiple origins of polyploidy in big sagebrush (*Artemisia tridentata* ; Asteraceae). *American J of Botany* **99**, 1962–1975 (2012).

10. L. P. Grossfurthner, E. R. Milano, P. A. Hohenlohe, L. P. Waits, B. A. Richardson, Population structure and hybridization under contemporary and future climates in a heteroploid foundational shrub species (Artemisia tridentata). *Front. Plant Sci.* **14**, 1155868 (2023).

11. S. E. Dreher, "Interspecific Hybridization in Sphaeralcea (Malvaceae)," The Claremont Graduate University. (2014).

12. T. L. Parchman, *et al.*, Genome-wide association genetics of an adaptive trait in lodgepole pine: ASSOCIATION MAPPING OF SEROTINY. *Molecular Ecology* **21**, 2991–3005 (2012).

13. B. K. Peterson, J. N. Weber, E. H. Kay, H. S. Fisher, H. E. Hoekstra, Double Digest RADseq: An Inexpensive Method for De Novo SNP Discovery and Genotyping in Model and Non-Model Species. *PLoS ONE* **7**, e37135 (2012).

14. J. B. Puritz, C. M. Hollenbeck, J. R. Gold, *dDocent* : a RADseq, variant-calling pipeline designed for population genomics of non-model organisms. *PeerJ* **2**, e431 (2014).

15. L. Fu, B. Niu, Z. Zhu, S. Wu, W. Li, CD-HIT: accelerated for clustering the next-generation sequencing data. *Bioinformatics* **28**, 3150–3152 (2012).

16. A. E. Melton, *et al.*, A draft genome provides hypotheses on drought tolerance in a keystone plant species in Western North America threatened by climate change. *Ecology and Evolution* **11**, 15417–15429 (2021).

17. A. E. Melton, *et al.*, A haploid pseudo-chromosome genome assembly for a keystone sagebrush species of western North American rangelands. *G3 Genes|Genomes|Genetics* **12**, jkac122 (2022).

18. H. Li, Aligning sequence reads, clone sequences and assembly contigs with BWA-MEM. [Preprint] (2013). Available at: http://arxiv.org/abs/1303.3997 [Accessed 10 October 2023].

19. H. Li, *et al.*, The Sequence Alignment/Map format and SAMtools. *Bioinformatics* **25**, 2078–2079 (2009).

20. E. A. Aronesty, ea-utils : "Command-line tools for processing biological sequencing data." (2011). Deposited 2011.

21. J. Catchen, P. A. Hohenlohe, S. Bassham, A. Amores, W. A. Cresko, Stacks: an analysis tool set for population genomics. *Molecular Ecology* **22**, 3124–3140 (2013).

22. N. C. Rochette, J. M. Catchen, Deriving genotypes from RAD-seq short-read data using Stacks. *Nat Protoc* **12**, 2640–2659 (2017).

23. J. R. Paris, J. R. Stevens, J. M. Catchen, Lost in parameter space: a road map for stacks. *Methods in Ecology and Evolution* **8**, 1360–1373 (2017).

24. P. Danecek, *et al.*, The variant call format and VCFtools. *Bioinformatics* **27**, 2156–2158 (2011).

25. A. Hapke, D. Thiele, GI b PS s: a toolkit for fast and accurate analyses of genotyping-by-sequencing data without a reference genome. *Molecular Ecology Resources* **16**, 979–990 (2016).

26. G. J. McKinney, R. K. Waples, C. E. Pascal, L. W. Seeb, J. E. Seeb, Resolving allele dosage in duplicated loci using genotyping-by-sequencing data: A path forward for population genetic analysis. *Molecular Ecology Resources* **18**, 570–579 (2018).

27. P. A. Hohenlohe, *et al.*, Genomic patterns of introgression in rainbow and westslope cutthroat trout illuminated by overlapping paired-end RAD sequencing. *Molecular Ecology* **22**, 3002–3013 (2013).

28. G. J. McKinney, R. K. Waples, L. W. Seeb, J. E. Seeb, Paralogs are revealed by proportion of heterozygotes and deviations in read ratios in genotyping-by-sequencing data from natural populations. *Molecular Ecology Resources* **17**, 656–669 (2017).

29. Z. Gompert, *et al.*, Admixture and the organization of genetic diversity in a butterfly species complex revealed through common and rare genetic variants. *Molecular Ecology* **23**, 4555–4573 (2014).

30. V. Shastry, *et al.*, Model-based genotype and ancestry estimation for potential hybrids with mixed-ploidy. *Molecular Ecology Resources* **21**, 1434–1451 (2021).

31. M. Nei, W. H. Li, Mathematical model for studying genetic variation in terms of restriction endonucleases. *Proc. Natl. Acad. Sci. U.S.A.* **76**, 5269–5273 (1979).

32. G. A. Watterson, On the number of segregating sites in genetical models without recombination. *Theoretical Population Biology* **7**, 256–276 (1975).

33. T. S. Korneliussen, I. Moltke, A. Albrechtsen, R. Nielsen, Calculation of Tajima's D and other neutrality test statistics from low depth next-generation sequencing data. *BMC Bioinformatics* **14**, 289 (2013).

34. T. S. Korneliussen, A. Albrechtsen, R. Nielsen, ANGSD: Analysis of Next Generation Sequencing Data. *BMC Bioinformatics* **15**, 356 (2014).

35. F. Tajima, Statistical method for testing the neutral mutation hypothesis by DNA polymorphism. *Genetics* **123**, 585–595 (1989).

36. J. Novembre, M. Stephens, Interpreting principal component analyses of spatial population genetic variation. *Nat Genet* **40**, 646–649 (2008).

37. G. McVean, A Genealogical Interpretation of Principal Components Analysis. *PLoS Genet* **5**, e1000686 (2009).

38. E. A. Sinclair, R. J. Hobbs, Sample Size Effects on Estimates of Population Genetic Structure: Implications for Ecological Restoration. *Restoration Ecology* **17**, 837–844 (2009).
